# Lead Increases Lipopolysaccharide-Induced Liver Injury through Tumor Necrosis Factor-α Overexpression by Monocytes/Macrophages: Role of Protein Kinase C and p42/44 Mitogen-Activated Protein Kinase

**DOI:** 10.1289/ehp.8550

**Published:** 2005-11-10

**Authors:** Yu-Jung Cheng, Bei-Chang Yang, Ming-Yie Liu

**Affiliations:** 1 Department of Environmental and Occupational Health,; 2 Institute of Basic Medical Sciences and; 3 Department of Microbiology and Immunology, National Cheng Kung University Medical College, Tainan, Taiwan

**Keywords:** lead, lipopolysaccharide, liver injury, monocytes/macrophage, p42/44 mitogen-activated protein kinase, protein kinase C, tumor necrosis factor-α

## Abstract

Although lead and lipopolysaccharide (LPS), both important environmental pollutants, activate cells through different receptors and participate in distinct upstream signaling pathways, Pb increases the amount of LPS-induced tumor necrosis factor-α (TNF-α). We examined the cells responsible for the excess production of Pb-increased LPS-induced TNF-α and liver injury, and the roles of protein kinase C (PKC) and p42/44 mitogen-activated protein kinase (MAPK) in the induction of TNF-α. Peritoneal injection of Pb alone (100 μmol/kg) or a low dose of LPS (5 mg/kg) did not affect serum TNF-α or liver functions in A/J mice. In contrast, coexposure to these noneffective doses of Pb plus LPS (Pb+LPS) strongly induced TNF-α expression and resulted in profound liver injury. Direct inhibition of TNF-α or functional inactivation of monocytes/macrophages significantly decreased the level of Pb+LPS-induced serum TNF-α and concurrently ameliorated liver injury. Pb+LPS coexposure stimulated the phosphorylation of p42/44 MAPK and the expression of TNF-α in CD14^+^ cells of cultured mouse whole blood, peritoneal macrophages, and RAW264.7 cells. Moreover, blocking PKC or MAPK effectively reduced Pb+LPS-induced TNF-α expression and liver injury. In summary, monocytes/macrophages were the cells primarily responsible for producing, through the PKC/MAPK pathway, the excess Pb-increased/LPS-induced TNF-α that caused liver injury.

Tumor necrosis factor-α (TNF-α) regulates a variety of biologic functions, including organ development, immune homeostasis, and malignance. The body subtly regulates the expression kinetics and dose of TNF-α to ensure its proper effect because TNF-α has opposite biologic effects in different circumstances (Aggarwal 2003; Pfeffer 2003). On the one hand, TNF-α is essential for the host in tissue repair and in protective immune responses against infection. On the other hand, inadequate TNF-α may have detrimental consequences in sepsis, tumor formation, and autoimmune diseases. Regulating the expression of TNF-α has been an important subject in managing acute inflammatory diseases that include bacterial sepsis (Spooner et al. 1992). Recent studies on chronic neuronal disease revealed a new feature of inflammation: a transient spike of TNF-α expression (i.e., a large amount that peaks after 1.5 hr and disappears after 3 hr) may induce neuronal degeneration resembling the delayed and progressive nature of the symptoms in patients with Parkinson’s disease (Gao et al. 2002). These findings indicate that the extent of TNF-α expression during a disease determines not only severity and survival rate but also delayed disease sequelae.

The *in vivo* lipopolysaccharide (LPS)-induced model of liver damage of mice, used to determine TNF-α–mediated organ failure, is both sensitive and convenient. LPS, a component of the outer membrane of gram-negative bacteria, plays a major role in inducing septic shock and is a potent inducer of TNF-α *in vivo* and *in vitro* (Goldfeld et al. 1990; Ulich et al. 1991). The binding of LPS to CD14/LPS-binding protein and Toll-like receptor-4 triggers multiple signal cascades that activate nuclear factor-κB and p42/44 mitogen-activated protein kinase (MAPK) and lead to the expression of proinflammatory cytokines, including TNF-α (Triantafilou and Triantafilou 2002). LPS causes liver injury at high doses (Kosai et al. 1999) but a modest, noninjurious inflammation at low doses (Ganey and Roth 2001) in several animal models. High-dose LPS-induced liver injury is partly attributed to excess TNF-α production (Hewett et al. 1993). TNF-α–associated signal transduction has been well characterized. TNF-α might trigger apoptosis in hepatocytes by signaling through the Fas-associated death-domain protein of the TNF receptor that activates caspases (Leist et al. 1996; Schuchmann et al. 2003). Blocking TNF production or trimming the signaling pathway using caspase-inhibitors reduces TNF-α–mediated liver injury (Kunstle et al. 1997). In addition, LPS induces apoptosis in macrophages through TNF-α (Comalada et al. 2003). Convincing evidence shows that metal pollutants in the living environment may modulate the effects of LPS. Among them, lead, an important industrial pollutant, not only altered the immune response (Luster et al. 1978) but also increased the mortality of an LPS challenge or bacterial infections in several animal studies (Dentener et al. 1989; Selye et al. 1966). Thus, the influence of environmental factors on TNF-α production is a significant issue.

Several pieces of evidence (Liu et al. 2001) suggest that Pb might act on calcium channels to alter intracellular calcium homeostasis in bone cells (Pounds 1984; Rosen and Pounds 1989; Schanne et al. 1989) and neuronal cells (Goldstein 1993; Pounds 1984; Rosen and Pounds 1989; Schanne et al. 1989). Although the cellular target of Pb is still elusive, exposure to Pb activates protein kinase C (PKC) in several types of cells, such as astrocytes and neuronal cells in the brain (Costa 1998; Markovac and Goldstein 1988). We previously (Cheng et al. 2004) demonstrated that Pb stimulates PKC to activate p42/44 MAPK, which results in the expression of TNF-α in glial cells. Although LPS and Pb trigger intra-cellular signals by different routes, Pb increases LPS-induced TNF-α production (Liu et al. 2005). Coexposure to Pb plus LPS also induces TNF-α expression through PKC and p42/44 MAPK, causing liver injury in rats (Cheng and Liu 2005). In this study, we measured the liver injury in mice as the biologic end point for exploring the mechanism of Pb-increased/LPS-induced TNF-α expression. Thus, the aims of this study were to identify the cells in the blood responsible for TNF-α release and to reveal the role of PKC and p42/44 MAPK in the induction of TNF-α during coexposure to Pb plus LPS.

## Materials and Methods

### Chemicals.

We obtained LPS (derived from *Escherichia coli*, serotype 055:B5), TNF-α inhibitor pentoxifylline (PTX), and macrophage cytotoxic agent GdCl_3–6_H_2_O (gadolinium chloride hexahydrate) from Sigma Chemical (St. Louis, MO, USA). Lead acetate was purchased from Merck (Darmstadt, Germany). PKC inhibitor chelerythrine chloride (C_21_H_18_NO_4_Cl) was obtained from Calbiochem (Bad Soden, Germany), and p42/44 MAPK inhibitor U0126 was purchased from Promega (Madison, WI, USA).

### Cells and animals.

Peritoneal macrophages were isolated by flushing the peritoneal cavity of A/J mice with 10 mL of sterile ice-cold phosphate buffer. Peritoneal lavage fluid was centrifuged at 200*g*, and pelleted cells were resuspended in Dulbecco’s modified Eagle medium (DMEM) supplemented with 10% fetal bovine serum (FBS), penicillin, and streptomycin (100 U/mL). Cells were then seeded at 2 × 10^5^/well in a 96-well plate, incubated at 37°C for 4 hr, and washed with phosphate-buffered saline (PBS) to remove unattached cells. Attached cells, taken as macrophages and confirmed with F4/80 stain, were used for sequential experiments. RAW264.7 cells, a mouse macrophage cell line (American Type Culture Collection, Rockville, MD, USA), were cultured in DMEM supplemented with 10% FBS. A/J mice weighing 20–25 g were obtained from and housed in the laboratory animal center of our institution. Animals were housed individually in a room with a 12/12-hr light/dark cycle and central air-conditioning (25°C, 70% humidity), and were fed with standard food *ad libitum*. The animal experiment procedures were reviewed and implemented through the Institutional Animal Care and Use Committee process of National Cheng Kung University.

### Blood collection and biochemistry study.

The blood of mice was collected from the inferior vena cava under ethyl ether anesthesia, drawn using venipuncture into serum separation tubes, allowed to clot for 10 min at room temperature, and then centrifuged (1,000*g*, 10 min, 4°C). Serum samples were stored at 70°C. To determine the serum concentrations of aspartate aminotransferase (AST) and alanine aminotransferase (ALT), serum was spotted to slides (Fuji Dri-Chem; Fujifilm, Kanagawa, Japan) and evaluated using a slide analyzer (Fuji Dri-Chem 3500S; Fujifilm).

### Preparation of mouse whole-blood and cytokine assays.

Induction of TNF-α in mouse whole blood was performed as described previously (Mullarkey et al. 2003). Briefly, Pb and LPS were added to heparinized whole blood obtained from A/J mice (100 μL/well). After 2 hr incubation at 37°C in a 5% CO_2_ atmosphere, the blood was centrifuged at 1,000*g* for 10 min at 4°C. TNF-α in conditioned medium was determined using enzyme-linked immunosorbent assay (ELISA) (R&D Systems, Minneapolis, MN, USA), measuring absorbance at 450 nm and extrapolating from a standard curve with a sensitivity limit of 32.5 pg/mL.

### Flow cytometric analysis.

To identify the TNF-α–secreting cells, Pb or LPS was added to whole blood with monensin (eBioscience, San Diego, CA, USA). Red blood cells were lysed using hypotonic shock, and leukocytes were subjected to surface CD14 labeling using phycoerythrin-conjugated CD14 antibody (eBioscience). Cells were fixed and per-meabilized using a commercial kit (Cytofix/Cytoperm; PharMingen, San Diego, CA, USA) and stained for intracellular TNF-α using fluorescein isothiocyanate (FITC)-conjugated rat anti-mouse TNF-α Ab (PharMingen). To analyze the phosphorylation status of p42/44 MAPK in peritoneal macrophages, exudate cells were fixed using 2% formaldehyde; they were then resuspended in methanol at a concentration of 90%. Cells were incubated with the primary phospho-p42/44 MAPK antibody (New England Biolabs, Beverly, MA, USA) for 30 min at room temperature. After being washed in PBS containing 0.5% FBS, secondary antibody hybridization was carried out using goat anti-rabbit IgG (Alexa Fluor 488; Molecular Probes, Eugene, OR, USA). Macrophage-specific marker F4/80 was first stained with biotin-conjugated anti-F4/80 antibody and then with phycoerythrin-conjugated streptavidin secondary antibody (eBioscience). Flow cytometric analysis was then performed (FACSCalibur; Becton Dickinson, San Jose, CA, USA). Data were analyzed using the CellQuest (BD Biosciences, San Jose, CA, USA) and WinMDI 2.8 software packages (University of Massachusetts, Amherst, MA, USA).

### Western blot analysis.

RAW264.7 cells were cultured in 0.01% FBS/DMEM for 30 min and sequentially stimulated with Pb (10 μM), LPS (1 ng/mL), Pb (10 μM) plus LPS (Pb+LPS) (1 ng/mL), or saline (control). After stimulation, cells were washed with cold PBS and then solubilized with ice-cold buffer containing 25 mM HEPES (pH 7.5), 300 mM NaCl, 1.5 mM MgCl_2_, 0.2 mM EDTA, 0.1% Triton X-100, 20 mM β-glycerophosphate, 0.1 mM sodium orthovanadate, 0.5 mM dithiothreitol, 100 μg/mL phenylmethylsulfonyl fluoride, and 2 μg/mL leupeptin. Approximately 10–30 μg of protein was separated using electrophoresis in a 10% sodium dodecyl sulfate–polyacrylamide gel. After electrophoresis, the protein was electro-transferred onto polyvinylidene fluoride membranes (NEN Life Science Products, Inc., Boston, MA, USA). Membranes were probed with antibodies specific for phospho-p42/44 MAPK or p42/44 MAPK (New England Biolabs). After being probed with a horseradish peroxidase–conjugated secondary antibody, protein signals were visualized using enhanced chemiluminescence reagents (Amersham, Arlington Heights, IL, USA) combined with Kodak X-ML film exposure (Eastman-Kodak, Rochester, NY, USA).

### Histology.

Liver tissue obtained from mice was fixed in 3.7% buffered formalin and embedded in paraffin. Sections (5 μm) were routinely stained with hematoxylin and eosin Y stain.

### Statistical analysis.

Data are expressed as mean ± SE. We used one-way or two-way analysis of variance and Student’s *t*-test. Statistical significance was set at *p* < 0.05.

## Results

### Expression of TNF-α and liver injury in mice after coexposure to Pb plus LPS.

**Figure 1:**
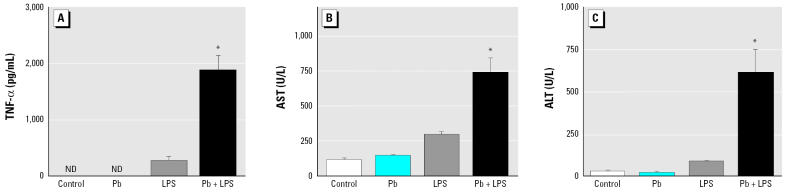
Expression of TNF-α (*A*), AST (*B*), and ALT (*C*) in A/J mice exposed to Pb, LPS, Pb+LPS, or saline (control). ND, not detectable. Blood was collected 1.5 hr after treatment to determine serum TNF-α (*A*). Serum AST (*B*) and ALT (*C*) were evaluated 24 hr posttreatment. *n* = 3 per treatment. *p < 0.05 compared with LPS.

**Figure 2:**
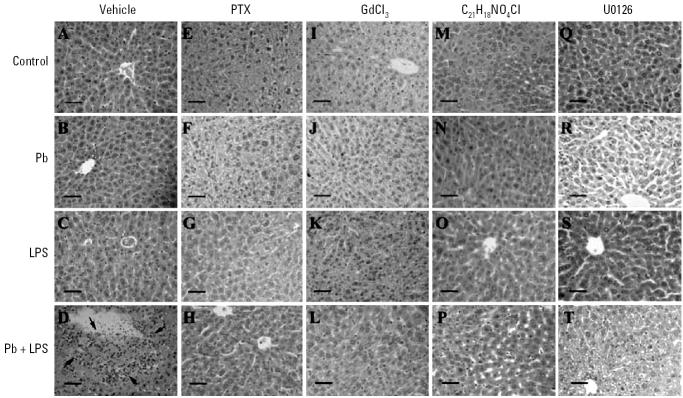
Histologic examination of liver damage in A/J mice challenged with Pb (*B*, *F*, *J*, *N*, *R*), LPS (*C*, *G*, *K*, *O*, *S*), Pb+LPS (*D*, *H*, *L*, *P*, *T*), or saline (control) (*A*, *E*, *I*, *M*, *Q*). To evaluate the effects of corresponding inhibitors, mice were pretreated with PTX (100 mg/kg) for 60 min (*E*, *F*, *G*, *H*), GdCl_3_ (40 mg/kg) for 24 hr (*I*, *J*, *K*, *L*), or C_21_H_18_NO_4_Cl (5 mg/kg) for 30 min (*M*, *N*, *O*, *P*), or U0126 (25 μmol/kg) for 10 min (*Q*, *R*, *S*, *T*). Mice were sacrificed after 24 hr. Arrows in (*D*) indicate the necrosis area. Tissue was stained with hematoxylin and eosin. Bars = 0.04 mm.

We evaluated the role of TNF-α in Pb-increased LPS-induced liver injury. A/J mice were intraperitoneally given Pb (100 μmol/kg) (Shinozuka et al. 1996), LPS (5 mg/kg) (Ohta and Sitkovsky 2001), Pb+LPS (Pb, 100 μmol/kg; LPS, 5 mg/kg), or saline (control). Blood was collected 1.5 hr later to determine serum TNF-α (Figure 1A) and 24 hr later to measure the levels of AST (Figure 1B) and ALT (Figure 1C) that indicate liver injury. Histologic examination of the liver was performed 24 hr posttreatment (Figure 2). Serum TNF-α was not detected in mice that received saline or Pb alone. Mice challenged with LPS alone showed a small amount of serum TNF-α (< 250 pg/mL) and a slight increase in AST (300 U/L) and ALT (30 U/L). However, we observed few histologic changes indicating hepatocellular damage in mice that received saline (Figure 2A), Pb alone (Figure 2B), or LPS alone (Figure 2C). Pb significantly increased LPS-induced TNF-α production in mice treated with Pb+LPS. The induction of serum TNF-α by LPS was drastically increased by Pb that reached around 2,000 pg/mL in 1.5 hr. Concurrently, the mean levels of AST and ALT were significantly elevated in the Pb+LPS group to 720 U/L and 600 U/L, respectively. In addition, Pb+LPS-treated mice showed multiple profoundly necrotic areas in the liver (Figure 2D).

**Figure 3:**
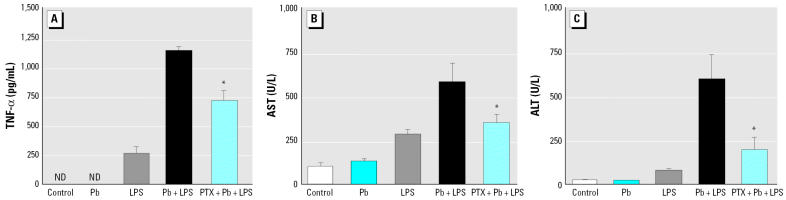
Effects of TNF-α inhibitor on liver damage in A/J mice intraperitoneally injected with PTX (100 mg/kg) or H_2_O as vehicle (control). One hour later, mice were stimulated with Pb, LPS, Pb+LPS, or saline only (control). ND, not detectable. Blood was collected either 1.5 hr after Pb, LPS, or Pb+LPS treatment to determine serum TNF-α (*A*), or 24 hr posttreatment to determine serum AST (*B*) and ALT (*C*). *n* = 3 per treatment. *p < 0.05 compared with Pb+LPS.

To establish a causal relationship between TNF-α and liver injury, we used PTX, a potent inhibitor of TNF transcription *in vivo* (Lechner et al. 1993), to suppress the production of TNF-α. Mice that had been given PTX (100 mg/kg) 1 hr before Pb+LPS treatment had lower serum TNF-α than did those that had not been given PTX (Figure 3A). Along with a decrease in TNF-α induction, the Pb+LPS-stimulated AST (Figure 3B) and ALT levels (Figure 3C) were also markedly attenuated by PTX. Moreover, PTX significantly decreased the number of necrotic hepatocellular lesions in Pb+LPS-treated mice (Figure 2H).

### TNF-α producing cells.

**Figure 4:**
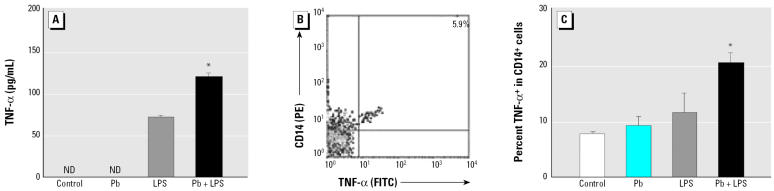
TNF-α expression in cultured whole blood of mice. Whole blood was stimulated *in vitro* with LPS (5 μg/mL) with or without Pb acetate (1 μM) for 1.5 hr. ND, not detectable. (*A*) TNF-α in serum measured by ELISA. (*B*) Density plot for blood cells from the Pb+LPS-treated group doubly stained for surface CD14 and intracellular TNF-α (*x*-axis, TNF-α; *y*-axis, CD14) and analyzed using flow cytometric analysis; the percentage shown is the ratio of TNF-α^+^ to CD14^+^ cells. (*C*) Mean percentage of TNF-α^+^ cells in CD14^+^ population (mean ± SE) of groups treated with Pb, LPS, or Pb + LPS (*n* = 3–5 per treatment). *p < 0.05 compared with LPS.

We detected rare TNF-α^+^ cells in the liver using immunohistochemical staining (data not shown). We then checked blood cells using an *in vitro* whole-blood culture model. LPS (5 μg/mL) increased the expression of mean TNF-α in whole-blood culture to 75 pg/mL (mean value) in 1.5 hr. Although Pb (1 μM) itself did not induce detectable TNF-α, it significantly increased the TNF-α–inducing effect of LPS, which then reached approximately 125 pg/mL (Figure 4A). Because CD14^+^ macrophages/monocytes have been documented as a major source of TNF-α (Haziot et al. 1996), we analyzed the CD14^+^ cells in the whole-blood culture after Pb+LPS coexposure. Using flow cytometric analysis, we determined whether CD14^+^ cells were the primary TNF-α–secreting cells in blood. The cells were stained with phycoerythrin–anti-CD14 and FITC-anti-TNF-α after stimulation. TNF-α^+^/CD14^+^ cells (5.9%) were separated from single negative cells (Figure 4B, top right quadrant). Intracellular TNF-α stain showed that < 8% of the CD14^+^ cells were in untreated or Pb-treated whole-blood culture. Approximately 12% of the CD14^+^ cells in the LPS group were also TNF-α^+^. Coexposure to Pb+LPS increased the number of TNF-α^+^ cells to about 20% of the CD14^+^ cells (Figure 4C).

### Macrophages/monocytes mediating Pb+LPS-induced liver injury.

**Figure 5:**
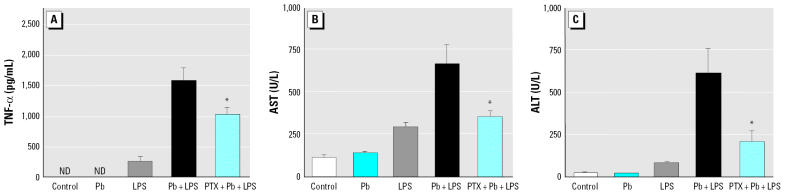
Liver damage after inactivating monocytes/macrophages in A/J mice intravenously injected with GdCl_3_ (40 mg/kg) or H_2_O as vehicle (control). After 24 hr, mice were stimulated with Pb, LPS, Pb+LPS, or saline only (control). ND, not detectable. Blood was collected either 1.5 hr posttreatment to determine serum TNF-α (*A*) or 24 hr posttreatment to determine serum AST (*B*) and ALT (*C*). *n* = 3 per treatment. *p < 0.05 compared with Pb+LPS.

To confirm whether Pb+LPS-induced liver injury involves macrophages/monocytes, we inactivated macrophages/monocytes using GdCl_3_ (40 mg/kg) 24 hr before Pb+LPS treatment. Mouse peritoneal macrophages that received GdCl_3_ were drastically reduced, and few cells expressed a high level of F4/80 (F4/80^high^), representing peritoneal macrophages in peritoneal exudate (data not shown). GdCl_3_ decreased the level of serum TNF-α in mice that had received Pb+LPS (Figure 5A). In parallel with a reduction in TNF-α, GdCl_3_ markedly decreased serum AST (Figure 5B) and ALT (Figure 5C) measured 24 hr after Pb+LPS treatment. GdCl_3_ also reduced the number of hepatocellular lesions in mice that received Pb+LPS, as shown by reduced necrotic areas in the liver (Figure 2L).

### The activation of p42/44 MAPK in peritoneal macrophages and RAW264.7 cells treated with Pb+LPS.

**Figure 6:**
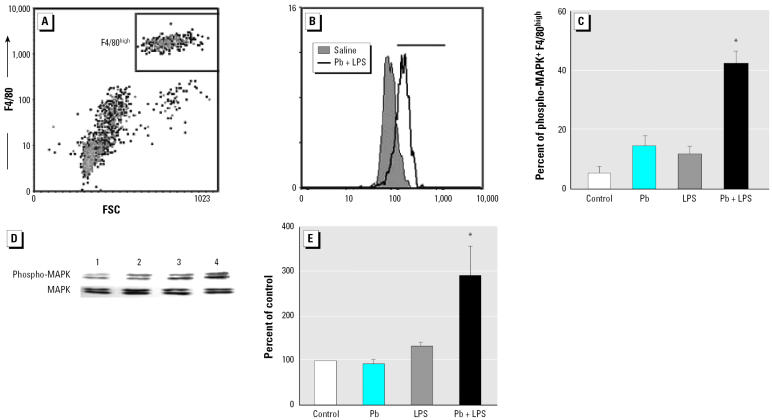
P42/44 MAPK phosphorylation in peritoneal macrophages and RAW264.7 cells. (*A*) Macrophages from peritoneal exudates were verified using F4/80 expression [*x*-axis, forward scatter (FSC); *y*-axis, F4/80]. (*B*) Representative histogram of intracellular phosphor-p42/44 MAPK staining in F4/80^high^ populations showing Pb+LPS and saline. (*C*) Mean percentage of phospho-p42/44^+^ cells in the F4/80^high^ population of groups treated with Pb, LPS, or Pb+LPS (mean ± SE, *n* = 3 per treatment). (*D*) RAW264.7 cells stimulated with Pb (10 μM, lane 2), LPS (1 ng/mL, lane 3), Pb (10 μM) plus LPS (1 ng/mL, lane 4), or saline (lane 1) for 5 min; total p42/44 MAPK and phosphorylated p42/44 MAPK were analyzed using Western blot analysis. (*E*) Relative intensities calculated by averaging three independent experiments (± SE). *Statistically significant from other treatment groups (*p* < 0.05).

We examined whether Pb acted through the common PKC and p42/44 MAPK pathway to increase LPS-induced TNF-α expression in peritoneal macrophages and RAW264.7 cells. The endothelial cells around large vessels in the livers of mice treated with Pb+LPS showed phosphorylated p42/44 MAPK, but the hepatocytes did not (data not shown). We further characterized the phosphorylation status of p42/44 MAPK in peritoneal macrophages, which showed high F4/80 expression (Figure 6A). An increase in the phosphorylation of p42/44 MAPK in peritoneal macrophages was observed in the Pb+LPS group (Figure 6B,C). Similarly, Pb+LPS treatment induced phosphorylation of p42/44 MAPK in RAW264.7 cells (Figure 6D,E).

### Effects of PKC and p42/44 MAPK inhibitors on TNF-α and liver injury in vivo.

**Figure 7:**
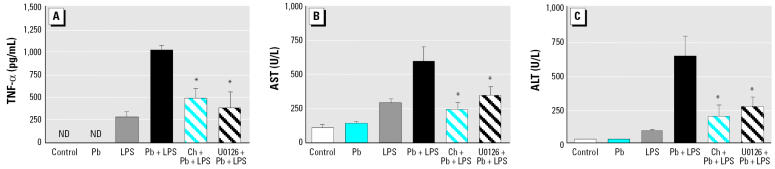
PKC and p42/44 MAPK as inhibitors on TNF-α expression and liver damage in mice pretreated (intraperitoneal injection) with C_21_H_18_NO_4_Cl (Ch; 5 mg/kg, 30 min), U0126 (25 μmol/kg, 10 min), or H_2_O vehicle (control, 30 min) and then stimulated with Pb, LPS, Pb+LPS, or saline only (control). ND, not detectable. Blood was collected either 1.5 hr after treatment to determine serum TNF-α (*A*), or 24 hr posttreatment to determine serum AST (*B*) and ALT (*C*). *n* = 3 per treatment. *p < 0.05 Ch+Pb+LPS or U0126+Pb+LPS compared with Pb+LPS.

To evaluate the actions of PKC and p42/44 MAPK *in vivo*, mice were treated with either C_21_H_18_NO_4_Cl (5 mg/kg) 30 min or U0126 (25 μmol/kg) 10 min before being exposed to Pb, LPS, or Pb+LPS. Both C_21_H_18_NO_4_Cl and U0126 effectively decreased the serum TNF-α induced by Pb+LPS (Figure 7A), as well as the AST and ALT levels elevated by Pb+LPS (Figure 7B,C). No obvious damage occurred in the livers of mice that had received C_21_H_18_NO_4_Cl or U0126 alone (Figure 2M,Q). Both C_21_H_18_NO_4_Cl and U0126 effectively attenuated the necrotic lesions developed in the livers of mice after Pb+LPS treatment (Figure 2P,T).

### PKC and p42/44 MAPK in the induction of TNF-α in peritoneal macrophages and RAW264.7 cells.

**Figure 8:**
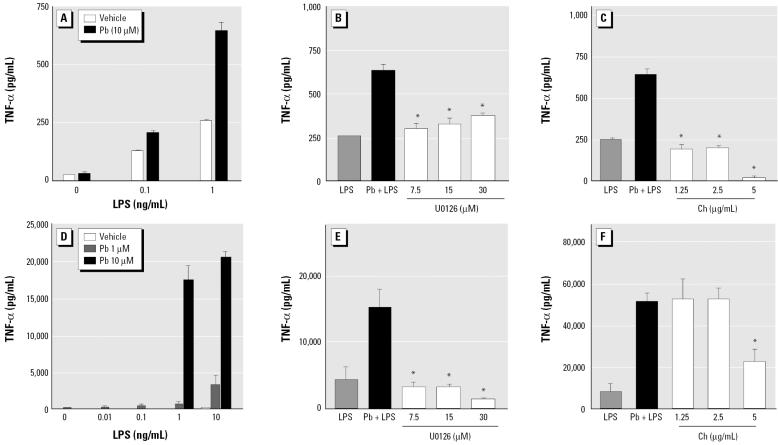
PKC and p42/44 MAPK in the induction of TNF-α in peritoneal macrophages and RAW264.7 cells. (*A*) TNF-α measured by ELISA in conditioned medium from peritoneal macrophages cultured in a 24-well plate (5 × 10^5^ cells/well). After 24 hr of attachment, cells were stimulated with LPS (0.1 or 1 ng/mL) combined with Pb (10 μM) for 3 hr. To evaluate the effect of kinase inhibitors, peritoneal macrophages were pretreated with U0126 (*B*) or C_21_H_18_NO_4_Cl (Ch) (*C*) for 30 min and then stimulated with 0.1 ng/mL LPS plus 10 μM Pb for 3 hr. (*D*) RAW264.7 cells were seeded in a 96-well plate (1 × 10^4^ cells/well) and stimulated for 3 hr with Pb (0, 1, or 10 μM) combined with LPS at various concentrations. To evaluate the effect of kinase inhibitors, RAW264.7 cells were pretreated with U0126 (*E*) or C_21_H_18_NO_4_Cl (*F*) for 30 min and then stimulated with 10 ng/mL LPS plus 10 μM Pb for 3 hr. *n* = 3. *p < 0.05 compared with Pb+LPS.

Pb (10 μM) significantly increased the expression of TNF-α induced by low doses of LPS (0.1 and 1 ng/mL) in mouse F4/80^high^ peritoneal macrophages (Figure 8A). In addition, Pb increased the expression of TNF-α in RAW264.7 cells after treatment with 1 or 10 ng/mL LPS (Figure 8D). This Pb-induced increase is more obvious at 10 μM than at 1 μM.

Using MAPK and PKC inhibitors, we demonstrated that p42/44 MAPK and PKC were involved in the Pb+LPS-induced TNF-α expression of peritoneal macrophages and RAW264.7 cells. At doses between 7.5 and 30 μM, U0126, a p42/44 MAPK inhibitor, significantly suppressed the Pb+LPS-induced expression of TNF-α. (Figure 8B,E). At a dose of 5 μg/mL, C_21_H_18_NO_4_Cl, a PKC inhibitor, also reduced Pb+LPS-associated TNF-α expression (Figure 8C,F).

## Discussion

Monocytes/macrophages are the primary secretors of TNF-α during inflammation and infection (Beutler and Cerami 1989). We demonstrated that the harmful effects of these cells triggered by LPS may become worse for the host in the presence of Pb, as reflected by severe liver injury. Inactivating the function of these phagocytic cells and blocking the signal pathways for TNF-α production effectively relieve the damage caused by environmental insults such as LPS and Pb.

Pb and low doses of LPS neither directly stimulated TNF-α^+^ production nor activated phosphorylation of MAPK in hepatocytes other than endothelial cells. Previous studies on the cellular source of TNF-α in animal exposed to Pb or LPS were not conclusive. Pb might increase the transcription of TNF-α mRNA in hepatocytes (Kubo et al. 1996), and LPS might stimulate liver Kupffer cells to release TNF-α (Suzuki et al. 1996; Tsukada et al. 2003). However, in a mixed culture of hepatocytes and Kupffer cells, Pb and LPS stimulated only a small increase in the production of TNF-α that did not cause obvious cell death in the cultured hepatocytes (Milosevic and Maier 2000; Zhang et al. 1996). In our animal model, serum TNF-α rapidly (within 1.5 hr) reached maximal levels after the Pb+LPS challenge. Moreover, cells releasing TNF-α after LPS stimulation *in vivo* have been identified as CD14^+^ (Haziot et al. 1996; Perera et al. 1997), but Kupffer cells express little CD14 (Lichtman et al. 1998). We also found that the TNF-α induced by Pb+LPS was expressed primarily by CD14^+^ cells in the whole-blood culture and in mouse F4/80^+^ peritoneal macrophages, which indicates that the TNF-α was produced by cells outside the liver. The increase in TNF-α caused by Pb in the serum of LPS-treated mice was approximately 10-fold. However, the increase of TNF-α was only 2-fold in peritoneal macrophages. This indicates that other cells and mechanisms might contribute to the Pb-increased LPS-induced TNF-α production *in vivo* and need further investigation.

A second line of evidence excluding hepatocytes as the source of TNF-α comes from studies of cell signaling. PKC and p42/44 MAPK are downstream signals of Pb stimulation in neurons (Olivi et al. 2003), bone-marrow–derived macrophages (Flohe et al. 2002), and glioma cells (Cheng et al. 2004), and they may regulate genes responding to Pb poisoning. Long-term exposure to Pb leads to PKC activation in rat livers; however, Pb also inhibited the activity of PKCα in a human hepatoma cell line (Liu et al. 1997; Tonner and Heiman 1997), and the inhibition was much more pronounced when Pb levels were high (Sun et al. 1999). Because circulating Pb will quickly deposit in the liver (Bornemann and Colburn 1985), we speculated (Cheng and Liu 2005) that a transient accumulation of Pb in the liver will suppress the activation of PKC and p42/44 MAPK in hepatocytes. Pb or LPS alone at the doses we used did not alter the phosphorylation of p42/44 MAPK in HepG2 cells (data not shown). In addition, in our animal model, we did not find phosphorylation of p42/44 MAPK in hepatocytes, further indicating that they were not induced to produce TNF-α in response to Pb exposure. In contrast to liver cells, coexposure to Pb+LPS significantly stimulated p42/44 MAPK phosphorylation in peritoneal macrophages; suppressing p42/44 MAPK phosphorylation or inhibiting PKC activity resulted in reduced TNF-α expression.

As mentioned above, both Pb and a high dose of LPS activated PKC and MAPK, which stimulated TNF-α expression (Cheng et al. 2004). In our study model, the synergistic effect of Pb+LPS was demonstrable: coexposing mice to Pb+LPS strongly induced TNF-α expression in peritoneal macrophages and in whole blood. In addition, significant interaction between the LPS-treatment factor and Pb-dose factor was indicated by dose-dependent TNF-α expression in different groups of RAW264.7 cells. Although Pb alone or LPS alone slightly increased the phosphorylation of MAPK in peritoneal macrophages, only coexposing them to Pb+LPS induced obvious p42/44 MAPK phosphorylation and TNF-α expression. Apparently, the minimal level of MAPK activation required to induce a large amount of TNF-α was higher than the levels of MAPK in cells after exposure to a single dose of Pb alone or LPS alone. After the signals initiated by Pb+LPS merged in the PKC and MAPK pathway, MAPK activation was sufficient for TNF-α production. That an otherwise insignificant effect of LPS in mice became vital after the mice had been coexposed to Pb+LPS has implications for setting the maximal tolerable concentration for a particular pollutant. It seems that risk assessment based on a single-exposure experimental design does not truly reflect the hazard of an environmental pollutant to humans.

In conclusion, the PKC/MAPK pathway leading to TNF-α expression played a key role in Pb+LPS-induced liver injury in mice. Our results also indicate that immune cells are very sensitive to environmental pollution and emphasize the synergistic effect of multiple pollutants in disease progression. Specifically, monocytes/macrophages may serve as watchful janitors in response to Pb+LPS, even when it injures their host.
